# Ulnar nerve entrapment syndrome: a special case report

**DOI:** 10.1186/s41065-025-00506-4

**Published:** 2025-07-14

**Authors:** Yifeng Han, Dachuan Sun, Mao Ye, Xindong Fan, Jiaxue Zhu

**Affiliations:** 1 Department of Interventional Therapy, Multidisciplinary Team of Vascular Anomalies, Shanghai Ninth People’s Hospital, Shanghai Jiao Tong University School of Medicine, Shanghai, PR China; 2 Department of Orthopedics, Shanghai Fengcheng Hospital, Shanghai Ninth People’s hospital, Shanghai Jiao Tong University School of Medicine, Shanghai, PR China

**Keywords:** Ulnar nerve entrapment syndrome, Arteriovenous malformations, Ilizarov technology, Rehabilitation

*Dear Editor*,

A 29-year-old female came to our clinic with the complaint of the lump at her left wrist, accompanied by pain, numbness and tingling in the forearm and the D3 and D4 fingers. The lump first onset since her puberty and then progressed during her pregnancy. In physical examination, a diffuse, hard, warm and pulsatile mass extended from the flexed side of the left wrist joint to the forearm with hypothenar atrophy, and could be moved readily with slight finger pressure (Fig. 1a). The extension of the middle and ring finger joint was limited (Fig. 1a). In radiology study, Magnetic Resonance Imaging (MRI) reveals flowing void effect in the lump of T2 (Fig. 1b). The patient went through surgical excision under the guidance of digital subtraction angiography (DSA), in order to precisely locate the arteriovenous malformation and the ulnar nerve. In the meanwhile, next-generation sequencing was performed in the lesion specimen. The genetic study revealed a somatic mutation in Map2K1(p.Gln56Pro) at mutation frequency of 3.66% (82/2237). The patient was finally diagnosed as ulnar nerve entrapment syndrome compressed by arteriovenous malformation.

**Fig. 1:**
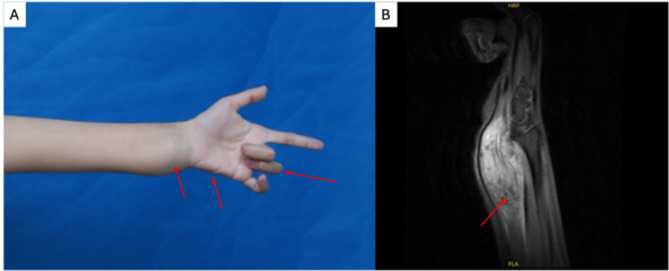
Clinical manifestation and Magnetic Resonance Imaging (MRI) of the lump at the wrist. **A**: Ulnar nerve entrapment: D3 and D4 extending limitation and hypothenar atrophy (the arrows). **B**: Flowing void effect in the lesion of T2 (the arrow)

Ulnar nerve entrapment syndrome is a compressive neuropathy that occurs when the ulnar nerve is trapped or compressed and can lead to progressive damage such as is pain, numbness, tingling in forearm and wrist [1]. Ulnar nerve entrapment syndrome could be caused by ulnar nerve damage itself or compression of other mass. Arteriovenous malformations (AVMs) are fast-flow vascular anomalies, consisting of abnormal artery‒vein shortcuts without intervening capillary network, and they constitute 4.7% of all vascular anomalies [2]. Recent years, the genetic study reveals that the AVM was caused by mutants in Ras/Raf/MAPK pathways, which may drive the development of AVMs [3–5]. Subsequent functional studies proposed that somatic KRAS mutations in endothelial cells (ECs) induced AVM-like phenomena in mice [6]. Above all, because of the hemodynamic effect as well as the biological feature of proliferation, angiogenesis, AVMs are progressive throughout life, and most of them begin during childhood and adolescence. In this case, the ulnar nerve entrapment syndrome occurs when the AVM progressed, infiltrated, and compressed where the ulnar nerve underlies.

Conventional treatments of AVM consist of embolization, resection, or a combination. The management of the arteriovenous malformation of wrist is challenging because the vascular, muscles, tendons, and nerves are all in the small space, where the multidisciplinary therapy such as interventional embolization, surgical excision, and rehabilitation for contracture of tendon and ultra nerve are needed. The main point of the management is radical excision of the arteriovenous malformation, tenolysis of the adhesion and tendon lengthening of extension tendons of D3/4. In this case, the patient went through radical surgical excision of under the guidance of digital subtraction angiography (DSA). Then the arteriovenous malformations were removed, and the tendons were released from the formal adhesion (Fig. 2a). Because of the formed contracture in ultra nerve as well as the extensor tendon, Illizarov technology was performed for tendon and nerve lengthening (Fig. 2b). The external fixation was removed 50 days later. During the four-month clinical follow-up, the patient showed no symptoms of ulnar nerve entrapment and satisfied with the movement of D3/4 fingers (Fig. 2c).

**Fig. 2:**
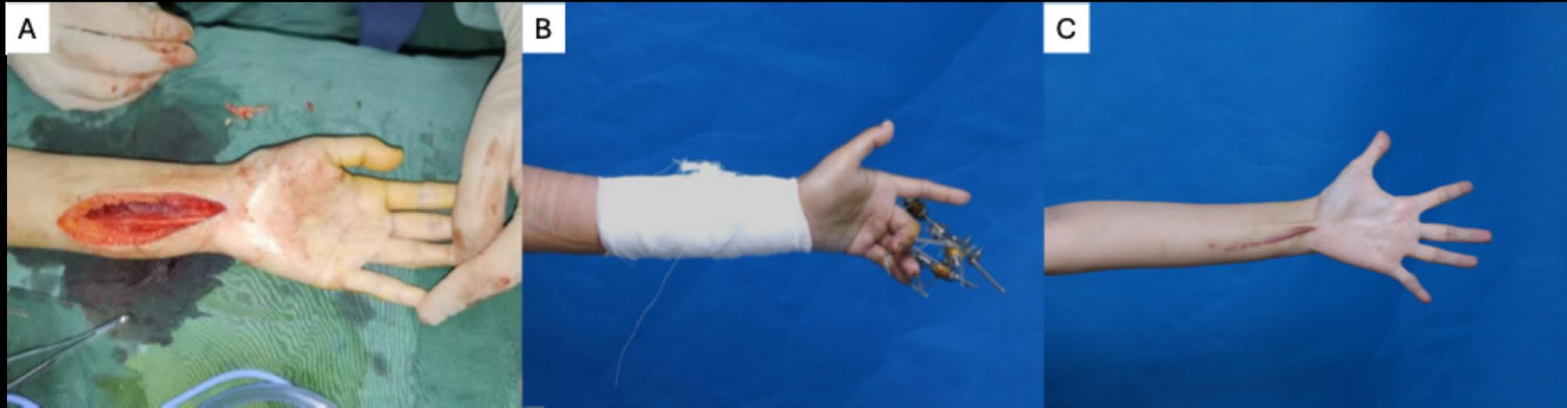
During the operation, 1-week post operation, 4-month follow up. **A**: The arteriovenous malformation was radically removed and tenolysis was performed. **B**: 1week after the operation, the contracture of the nerve and tendons were lengthened by Illizarov Technology after radical excision and tenolysis. **C**: 4 month after the operation, no symptoms of ulnar nerve entrapment syndrome was showed. D3 and D4 finger movement were in full range

In this case, we report a special clinical case of ulnar nerve entrapment syndrome caused by arteriovenous malformation who should be managed by multidisciplinary team, where interventional embolization, radical surgical excision, and rehabilitation by Illizarov technology will bring a good outcome.

## Data Availability

No datasets were generated or analysed during the current study.
